# Access to Healthcare for Appendicitis Patients in the United States Based on Insurance Coverage

**DOI:** 10.7759/cureus.70699

**Published:** 2024-10-02

**Authors:** My Yen Tran, Nirmal Patel, Abdallah Bakeer, Reshma S Kumar

**Affiliations:** 1 Surgery, Wroclaw Medical University, Wroclaw, POL; 2 Internal Medicine, St. George’s University School of Medicine, St. George's, GRD; 3 Internal Medicine, Jordan University of Science and Technology, Irbid, JOR

**Keywords:** appendicitis, cigna, healthcare access disparity, medicaid, waiting times

## Abstract

Introduction: Health insurance plays a crucial role in ensuring access to healthcare services, addressing affordability concerns. Insurance options have varying wait times and acceptance challenges, which can greatly impact patients in emergency conditions, such as appendicitis. This study aims to find out the healthcare access disparities for appendicitis patients in states with low Medicaid coverage.

Methods: A three-week virtual cross-sectional study (Oct 20-Nov 10, 2023) evaluated healthcare accessibility for appendicitis symptoms in North Dakota, Utah, Wyoming, and New Hampshire. Using www.healthgrades.com, data on general surgeons within 10 miles prioritizing new patients with three stars or above were collected and then analyzed with StataCorp 2023.

Results: The study was done among 81 physicians among whom, 61 (75.31%) were male and 20 (24.69%) were female, mostly MD (76, 93.83%), DO (3, 3.70%), and APRN (2, 2.47%). Cigna (n=77) is mostly accepted by more physicians than Medicaid (n=33) across the states. The average waiting period for Cigna (9.45±10.54 days) is mostly longer than Medicaid (4.71± 9.63 days). Our findings of P-values >0.05 reveal no significant associations in insurance acceptance or waiting times.

Conclusions: Although varying wait times were observed across states, no significant disparities were found in appointment waiting periods based on insurance types.

## Introduction

Health insurance is an important aspect of modern healthcare as it provides people with access to essential medical services while ensuring financial security [1,2]. In the United States, health insurance is categorized into two main types: private and government-backed. Private insurance, often provided through employers, offers a range of plans with diverse coverage options, exemplified by providers such as Cigna. Conversely, government initiatives like Medicaid are tailored to support individuals with lower incomes, playing a vital role in extending healthcare access to vulnerable populations.

Within this framework, timely access to healthcare is particularly critical for conditions such as appendicitis, a common surgical emergency. Delays in diagnosis and treatment can escalate the risk of severe complications, including appendiceal rupture, peritonitis, and sepsis, underscoring the urgency of prompt intervention [3]. Recent research has shed light on disparities in healthcare access related to insurance type. For instance, studies by Decker in 2011 revealed that a significant proportion of physicians were hesitant to accept new Medicaid patients, highlighting potential barriers faced by individuals relying on government-backed insurance [4]. Similarly, investigations by Gotlieb in 2021 found that Medicaid patients often experience longer waiting times for appointments compared to those with commercial insurance coverage [5].

By elucidating the relationship between health insurance type and access to care for appendicitis, this study seeks to provide evidence-based insights that can inform policy decisions and healthcare practices. By addressing any disparities in access to care, healthcare systems can strive to ensure equitable treatment for all patients, regardless of their insurance status. Ultimately, this research contributes to the broader goal of enhancing healthcare access and quality, thereby improving patient outcomes and overall healthcare system performance.

Aims and objectives

The study aims to investigate the influence of health insurance type on access to healthcare services, specifically focusing on patients presenting with appendicitis as a time-sensitive surgical emergency. The study aims to examine disparities in access to care between individuals with private insurance and those covered by government-backed programs such as Medicaid.

## Materials and methods

A cross-sectional study of accessibility to healthcare in the United States was conducted virtually over a three-week period (October 20th-November 10th 2023). This study, gathering solely organizational data on insurance acceptance rates and time to appointment through a phone call survey, did not meet the criteria for "human subject" research, thus exempting it from the need for institutional review board approval or informed consent. This article adheres to the STROBE checklist for cross-sectional studies, ensuring transparency and rigor in reporting methodology and findings [6].

The authors first identified states with the lowest Medicaid coverage via sources including the official Medicaid and CHIP website and Medicaid State Fact Sheets provided by the Kaiser Family Foundation [7,8]. After identifying North Dakota, Utah, Wyoming, and New Hampshire as states with low Medicaid coverage, the three most populated cities within each state were determined. This selection process involved referencing demographic data from Demographics.com, resulting in the identification of the following cities: Fargo, Bismarck, and Grand Forks for North Dakota; Salt Lake City, West Valley City, and West Jordan for Utah; Cheyenne, Casper, and Gillette for Wyoming; and Manchester, Nashua, and Concord for New Hampshire [9].

Subsequently, a comprehensive list of healthcare providers operating within the selected cities was generated using HealthGrades [10]. The inclusion criteria within this list were general surgeons who practiced in the cities of interest, taking new patients into their practice, working within a 10-mile radius with a rating of three stars or above. Retired physicians, permanently closed offices, offices not accepting new patients, incorrect contact information provided, or unreachable by phone were excluded from the study. In case of limitations to contact with physician offices such as reaching a voicemail directly or being placed on hold for longer than 10 minutes, the office was contacted on two separate occasions and ultimately excluded from the study. Furthermore, the type of insurance plan was mentioned as employer-provided, if asked. Finally, if the offices needed further information about the scenario provided, the data collection for a study was mentioned specifically to avoid taking away valuable time from patients. Physicians with incomplete information were excluded from the analysis.

Data collection included several parameters for each healthcare practitioner, including demographic information such as gender determined by photograph, state, and city. Questions regarding health insurance acceptance were asked, including whether Medicaid and Cigna health insurance were accepted, the time for the next available appointment based on insurance, and whether new patients were accepted based on insurance status. The data was collected by the authors by calling up the physician's offices with a case scenario for a patient exhibiting initial symptoms of appendicitis:“A 31-year-old male with pain in lower right abdomen since the past 2 days presenting to the surgery clinic for evaluation.”

Efforts to address potential sources of bias included systematically excluding physicians who did not meet the inclusion criteria and ensuring consistency in data collection methods. The study size was determined based on the availability of general surgeons meeting the inclusion criteria within the selected geographic areas.

The data was compiled on an Excel file (Microsoft, Redmond, WA) and StataCorp 2023 (Stata Statistical Software: release 18. College Station, TX: StataCorp LLC) was used to perform statistical model analysis. Statistical methods included descriptive analysis of demographic characteristics and comparative analysis of healthcare access based on insurance coverage.

## Results

**Figure 1:**
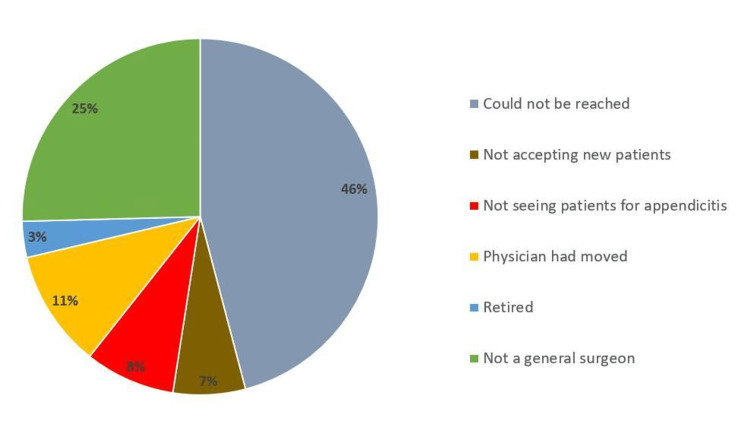
Reasons for exclusion of healthcare providers from the study.

The study initially screened 203 physicians practicing within a 10-mile radius of key cities in North Dakota, Utah, Wyoming, and New Hampshire. After applying exclusion criteria, 122 physicians were excluded from the study, leaving 81 physicians included for analysis. Figure 1 shows the reasons for exclusion with 56 (46%) of those physicians not being reachable, 31 (25%) of them not being general surgeons as stated otherwise online, and 13 (11%) having moved. 

The characteristics of healthcare providers in Table 1 show 61 (75.31%) male physicians participating, whereas female physicians were 20 (24.69%). Most physicians held MD degrees (76, 93.83%), while three (3.70%) held DO degrees, and two (2.47%) were APRNs. Five stars had been given to 40 (49.38%) physicians, four stars to 33 (40.74%), and three stars to 8 (9.88%). Four states were analyzed, with 34 (41.98%) physicians residing in Utah, 28 (34.57%) being in North Dakota, 13 (16.05%) in New Hampshire, and six (7.41%) working in Wyoming. 

**Table 1:** Characteristics of healthcare providers. Values are written in n (%).

Variables	No (%)
Gender	
Male	61 (75.31)
Female	20 (24.69)
Designation	
MD	76 (93.83)
DO	3 (3.70)
APRN	2 (2.47)
Star/rating	
5 stars	40 (49.38)
4 stars	33 (40.74)
3 stars	8 (9.88)
State	
New Hampshire	13 (16.05)
North Dakota	28 (34.57)
Utah	34 (41.98)
Wyoming	6 (7.41)

Table 2 shows the number of healthcare providers accepting Medicaid and Cigna. Of the 33 healthcare professionals that accept Medicaid, eight (24.24%) are from New Hampshire, one (3.03%) is from North Dakota, 18 (54.55%) from Utah, and six (18.18%) are from Wyoming. Cigna was accepted by 77 healthcare professionals with 13 (16.88%) being from New Hampshire, 26 (33.77%) from North Dakota, 32 (41.56%) from Utah, and six (7.79%) from Wyoming. Cigna was accepted by more healthcare professionals than Medicaid in New Hampshire, North Dakota, and Utah, whereas in Wyoming the number of healthcare professionals accepting both health insurances was the same number. The P-value for North Dakota corresponds to 0.77 and for Utah, it is 0.12, which gives a total P-value of 0.08 that is all above the P-value of 0.05. Therefore, our results suggest no significant association between healthcare providers accepting Medicaid and Cigna insurance. Table 3 shows the average waiting days based on the type of insurance.

**Table 2:** Healthcare providers accepting Medicaid and Cigna. Values are mentioned in n (%); test used: Pearson’s chi-square test. P-value <0.05 is considered significant.

State	Medicaid	Cigna	P-value
New Hampshire	8 (24.24)	13 (16.88)	-
North Dakota	1 (3.03)	26 (33.77)	0.77
Utah	18 (54.55)	32 (41.56)	0.12
Wyoming	6 (18.18)	6 (7.79)	-
Total	33	77	0.08

**Table 3:** Average waiting days based on type of insurance. Values are mentioned in mean and standard deviation. Test used: Kruskal-Wallis test. P-value <0.05 is considered significant.

State	Medicaid (n=33)	Cigna (n=77)	P-value
New Hampshire	14.61±18.63	26.15±14.58	0.12
North Dakota	0.35±0.18	3.60±4.56	0.79
Utah	3.58±4.27	7.52±4.23	0.85
Wyoming	11.5±9.48	11.5±9.48	0.41
Total	4.71±9.63	9.45±10.54	0.21

In New Hampshire, the waiting period for Medicaid was 14.61±18.63 days, and for Cigna, 26.15±14.58 days, which gives a P-value of 0.12. The waiting period in North Dakota was 0.35±0.18 days for Medicaid and 3.60±4.56 days for Cigna, giving a P-value of 0.79. For Utah, the P-value was 0.85, which comes from a waiting period of 3.58±4.27 days for Medicaid and 7.52±4.23 days for Cigna. When it comes to Wyoming, the waiting period for Medicaid and Cigna was both the same with 11.5±9.48 days, leading to a P-value of 0.41. The total waiting period for Medicaid was 4.71±9.63 days and for Cigna, 9.45±10.54 days, resulting in a P-value of 0.21.

Patients insured with Cigna had to wait longer in New Hampshire, North Dakota, and Utah for an appointment, whereas in Wyoming the results were the same with both Cigna and Medicaid. With the P-values all above 0.05, our results suggest no significant association between average waiting days based on the type of insurance.

## Discussion

The study aimed to assess healthcare access disparities for appendicitis patients based on insurance coverage, focusing on Cigna and Medicaid, in states with low Medicaid coverage. Key results indicate that while there were variations in wait times for appointments across states, no significant disparities were found based on insurance types. Both Cigna and Medicaid were accepted by healthcare providers, with Cigna being slightly more widely accepted. However, the difference in average waiting times between the two insurance types was not statistically significant.

In America, health insurance is a common means of financing one’s healthcare expenses by providing coverage and access to care, lowering the high costs of medical treatments, and providing stability to patients and their families [11]. The majority of Americans have private health insurance coverage that is obtained through employment, direct purchase, or Tricare, such as Cigna. Some Americans, however, are eligible to receive coverage offered by the government such as Medicare, Medicaid, and Champva/VA if they are unable to get it privately. Our study determined there to be no difference in acceptance of healthcare coverage between healthcare providers. Disparities in healthcare access based on insurance can potentially have a negative and life-altering impact on patient care. One study found that patients without reliable health insurance were overdue for colorectal cancer screening (P=0.04) [12]. These findings show how even late-stage cancer could have been treated effectively if a diagnosis had been made earlier and there were no barriers to healthcare created by access to insurance.

The average wait time for a patient to see a physician after making an appointment in America is 26 days, which is an 8% increase from the year 2017 [13]. One study found that health insurance had an effect on patient wait times. Patients who did not have health insurance or access to other resources had to wait for federal evaluations for their low-income status to see if they qualified for governmental coverage, like Medicare, and this led to delays in seeing a physician even if it was only to be prescribed medication [14]. Our study determined there to be no difference in wait times based on the health insurance type. This was not the case, however, in a study of wait times to see a primary care physician based on health insurance. The study compared wait times for patients who had either simulated Medicare or private insurance coverage and found that those with simulated Medicare had to wait 1.3 days longer than those with private insurance [5]. Similar findings were also observed in a cross-sectional study that looked at insurance acceptance and wait times to see a dermatologist, which found that patients with Medicaid had significantly longer wait times and difficulty securing an appointment compared to patients with private insurance [15].

Limitations

One limitation of this study is that only HealthGrades was used to assess information on doctors in the cities, which limited the information available for use in the study. This limitation could have been reduced by using other databases along with HealthGrades. Another limitation of the study is that healthcare professionals of all ratings were not included, such as one star and two stars, and should have because those professionals were present in the cities of study irrespective of their rating. Another factor of the study that was not accounted for was acknowledging a possible difference in reaction from the clinic if it were an actual patient experience as opposed to a case scenario about appendicitis. Finally, more states could have been included in the study instead of only including the four states with the lowest Medicaid coverage, which may have conferred a bias not applicable to the entire country.

Given the limitations of the study, a cautious interpretation of the results is warranted. While the findings suggest no significant disparities in healthcare access based on insurance coverage for appendicitis patients in the selected states, the results should be interpreted within the context of the study design and limitations. The lack of statistical significance in wait times may be influenced by the small sample size and the inherent variability in healthcare practices across different regions. It is important to consider these factors when drawing conclusions from the study.

The generalizability of the study results may be limited by the specific geographic locations and the focus on states with low Medicaid coverage. Additionally, the reliance on data from www.healthgrades.com may not capture the full spectrum of healthcare providers in these states. Therefore, caution should be exercised when extrapolating the findings to other regions or populations. Further research with larger sample sizes and more comprehensive data collection methods is needed to improve the generalizability of the findings and provide a more robust understanding of healthcare access disparities for appendicitis patients based on insurance coverage.

## Conclusions

This study examines healthcare accessibility for appendicitis patients in states with low Medicaid enrollment, focusing on insurance coverage. While variations in wait times were observed, no significant disparities between Cigna and Medicaid were found. Despite the lack of statistical significance, the findings highlight the need to address potential barriers for vulnerable populations relying on Medicaid.

Expanding the study to a larger population would better confirm these findings. Authorities should address healthcare disparities and employ more doctors to reduce patient wait times.
